# An unexpected gastric submucosal mass-like lesion

**DOI:** 10.1055/a-2183-6060

**Published:** 2023-10-27

**Authors:** Yue Hu, Liang Huang, Cheng Ye, Yi Xu, Bin Lu

**Affiliations:** 1 Department of Gastroenterology, The First Affiliated Hospital of Zhejiang Chinese Medical University, Zhejiang Provincial Hospital of Chinese Medicine, Zhejiang, China; 2 Key Laboratory of Digestive Pathophysiology of Zhejiang Province, The First Affiliated Hospital of Zhejiang Chinese Medical University, Zhejiang, China

A 70-year-old man was referred to our hospital with a gastric antrum mass ( Fig. 1 ) incidentally found during a screening endoscopy. After his admission, an abdominal enhanced computed tomography (CT) scan was performed, which showed a thickening of the gastric wall in the gastric antrum with a mixed-density shadow ( Fig. 2 ). In this context, the patient was submitted to endoscopic ultrasonography and there was a heterogeneous echo with the obscure boundary of the antrum originating from the submucosal layer ( Fig. 3 ). Follow-up or surgical operation was advised owing to the poorly circumscribed lesions, but the patient requested an endoscopic resection to further clarify the nature of this mass.

**Fig. 1:**
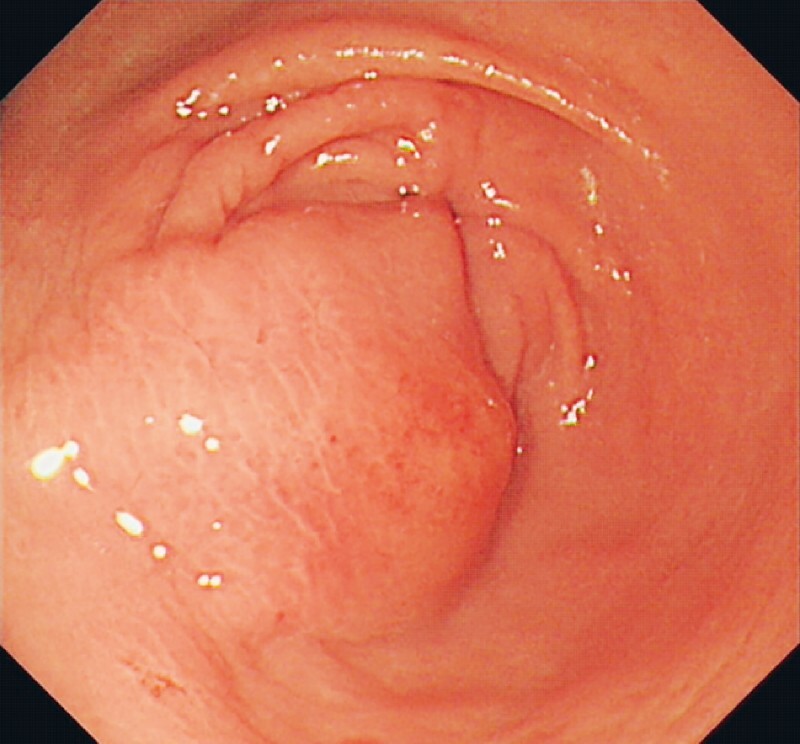
Endoscopic view of a gastric antrum submucosal lesion.

**Fig. 2:**
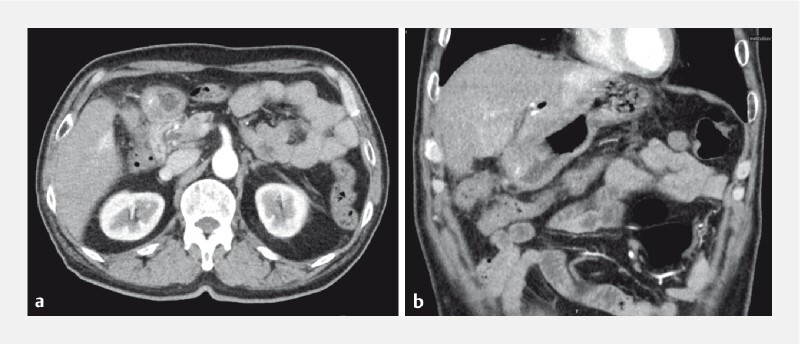
Computed tomography images of a gastric antrum submucosal lesion. **a** Transverse plane. **b** Coronal plane.

**Fig. 3:**
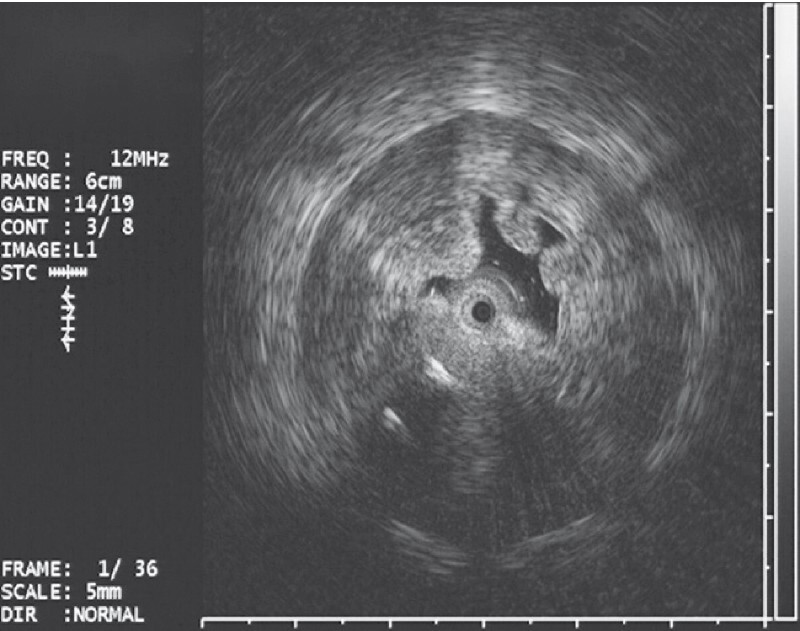
Endoscopic ultrasonography images of a gastric antrum submucosal lesion.

The procedure was performed with the patient under endotracheal intubation and general anesthesia. An incision was made along the incisura lesion of the gastric antrum after coagulation marking on the lesion margin, and there was no evidence of the obvious tumor body. Suddenly, a white foreign body appeared when cutting along the surface of the muscle layer ( Fig. 4 ), which was removed and then retrieved using a snare. Subsequently, the mass-like lesion was fully resected, and the wound was treated with hot coagulation forceps ( Video 1 ). On close inspection of the foreign body, it was a plastic clip similar to a Hem-o-lok clip ( Fig. 5 ). In a review of the patient’s surgical history, the Hem-o-lok clip might have been used in the previous laparoscopic cholecystectomy 2 years previous.

**Fig. 4:**
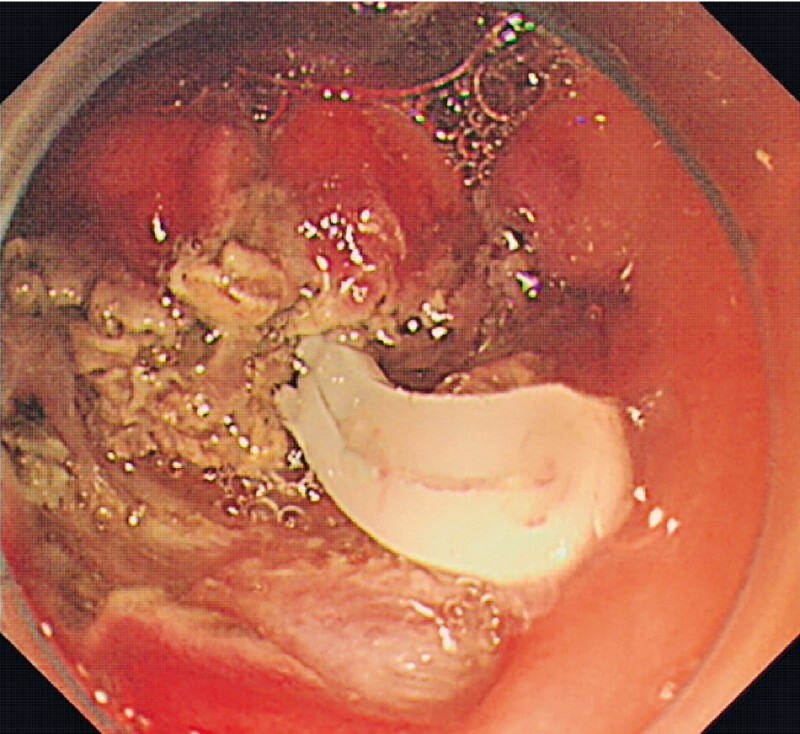
Endoscopic view showing a white foreign body after incision of the muscle layer.

**Video 1** An unexpected gastric submucosal mass-like lesion.


**Fig. 5:**
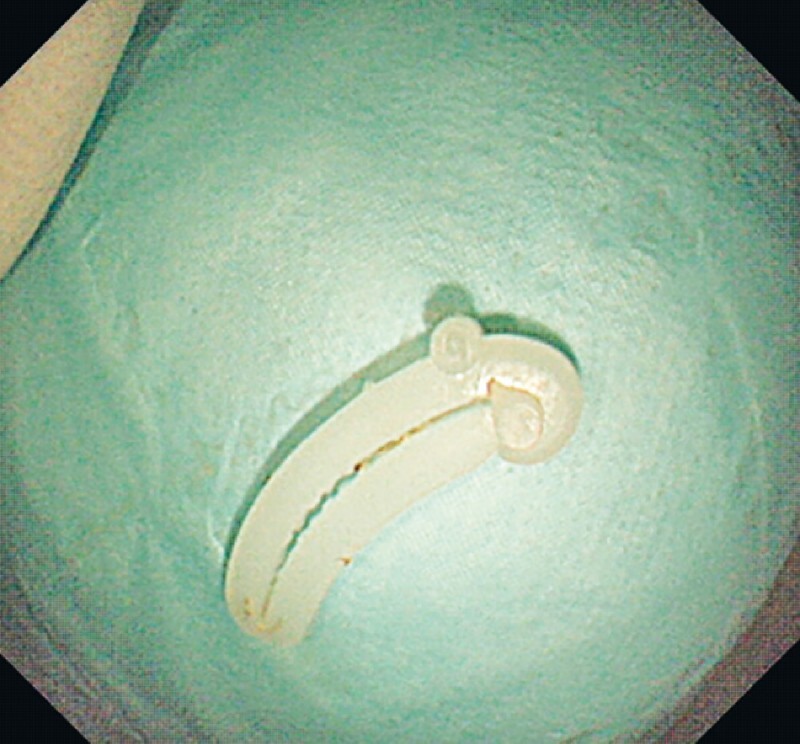
The final view of the foreign body (Hem-o-lok clip).

The patient remained well and was discharged after 4 days without complications. The final pathological examination of the mass revealed a reactive nodular fibrous pseudotumor.

The Hem-o-lok clip is used frequently during laparoscopic procedures, and a few case reports of clip migration have been published 1 2 3 4 . In our case, the patient was asymptomatic and the clip was found in an endoscopic resection of the mass-like lesion, which is a rare report.

Endoscopy_UCTN_Code_CCL_1AB_2AD_3AD

## Notes

Endoscopy E-Videos

https://eref.thieme.de/e-videos
*E-Videos* is an open access online section of the journal *Endoscopy* , reporting on interesting cases and new techniques in gastroenterological endoscopy. All papers include a high-quality video and are published with a Creative Commons CC-BY license. Endoscopy E-Videos qualify for HINARI discounts and waivers and eligibility is automatically checked during the submission process. We grant 100% waivers to articles whose corresponding authors are based in Group A countries and 50% waivers to those who are based in Group B countries as classified by Research4Life (see: https://www.research4life.org/access/eligibility/ ). This section has its own submission website at https://mc.manuscriptcentral.com/e-videos
